# Exosomal miR-140-5p inhibits osteogenesis by targeting IGF1R and regulating the mTOR pathway in ossification of the posterior longitudinal ligament

**DOI:** 10.1186/s12951-022-01655-8

**Published:** 2022-10-15

**Authors:** Yifan Tang, Yanqing Sun, Junkai Zeng, Bo Yuan, Yin Zhao, Xiangwu Geng, Lianshun Jia, Shengyuan Zhou, Xiongsheng Chen

**Affiliations:** Spine Center, Department of Orthopedics, Shanghai Changzheng Hospital, Second Military Medical University, 415 Fengyang Road, Shanghai, 200003 China

**Keywords:** OPLL, miR-140-5p, MSC, Exosome

## Abstract

**Background:**

Ossification of the posterior longitudinal ligament (OPLL) is a disabling disease whose pathogenesis is still unclear, and there are no effective cures or prevention methods. Exosomal miRNA plays an important role in the osteogenesis of ectopic bone. Therefore, we focused on the downregulation of miR-140-5p in OPLL cell-derived exosomes to explore the mechanism by which exosomal miR-140-5p inhibits osteogenesis in OPLL.

**Results:**

Exosomes were isolated by differential centrifugation and identified by transmission electron microscopy, nanoparticle tracking analysis, and exosomal markers. Exosomal RNA was extracted to perform miRNA sequencing and disclose the differentially expressed miRNAs, among which miR-140-5p was significantly downregulated. Confocal microscopy was used to trace the exosomal miR-140-5p delivered from OPLL cells to human mesenchymal stem cells (hMSCs). In vitro, we verified that exosomal miR-140-5p inhibited the osteoblast differentiation of hMSCs by targeting IGF1R and suppressing the phosphorylation of the IRS1/PI3K/Akt/mTOR pathway. In vivo, we verified that exosomal miR-140-5p inhibited ectopic bone formation in mice as assessed by micro-CT and immunohistochemistry*.*

**Conclusions:**

We found that exosomal miR-140-5p could inhibit the osteogenic differentiation of hMSCs by targeting IGF1R and regulating the mTOR pathway, prompting a further potential means of drug treatment and a possible target for molecular therapy of OPLL.

**Supplementary Information:**

The online version contains supplementary material available at 10.1186/s12951-022-01655-8.

## Introduction

Ossification of the posterior longitudinal ligament (OPLL) is a kind of degenerative disease in which heterotopic ossification in the posterior longitudinal ligament compresses the spinal cord and nerve roots, produces a series of clinical manifestations, and leads to dysfunction of the spinal cord. OPLL occurs mostly in the cervical spine (less often in the thoracic and lumbar spine) and can be accompanied by diffuse idiopathic skeletal hyperostosis [1–3]. OPLL has a high incidence in Japan and other East Asian countries; therefore, OPLL has also been called "Japanese disease" [4]. A study in Japan showed that the prevalence of OPLL in people over 30 years old is 1.9% to 4.3% [5]. The prevalence in other East Asian countries is similar. The average prevalence in Chinese individuals is 3.08% [6], but the prevalence in Koreans is lower, approximately 0.6% [7]. The prevalence of OPLL in the United States and Europe is only 0.01% to 1.7% [4]. The main symptoms of OPLL include limb movement and sensory disorders and sphincter function damage. The continued progression of the disease may lead to paraplegia, severely affecting patients’ quality of life and increasing family and socioeconomic burdens.

However, the cause of OPLL is still unclear. To investigate the cause, mesenchymal stem cells were isolated from human spinal ligaments, including the posterior longitudinal ligament, and studied [8, 9]. OPLL has the characteristic of family inheritance, so most scholars believe that the combination of genetics, environment, and other factors together lead to osteogenic differentiation of human mesenchymal stem cells (hMSCs), resulting in the occurrence of OPLL, but it is still at the stage of speculation and theory [10–12].

The pathological mechanism of OPLL is probably related to changes in a variety of extracellular microenvironmental regulatory substances, which induce the osteogenic differentiation of hMSCs. Extracellular microvesicles (EVs) are effective microenvironmental regulatory components and potential biomarkers. The various vesicles secreted by cells can be distinguished by their diameters. Exosomes, with a diameter of 50 nm-150 nm, are considered to be active substances with strong biological effects [13, 14]. Exosomes are generally released through multivesicular exocytosis and participate in intercellular signal transduction. They can deliver a series of signal transduction proteins, mRNAs and microRNAs (miRNAs, miR) to exert biological effects, participating in the occurrence and development of many diseases, including atherosclerosis, coronary artery disease, hematological diseases, diabetes, and cancer [15–17]. Many miRNAs are abnormally expressed in (Additional file 4: Table S1) diseased tissues, and some miRNAs, as important biomarkers, are important indicators for the diagnosis and prognosis of diseases. Studies have found that many miRNAs are delivered from cell to cell in the form of vesicles. Due to the protection of vesicles, these miRNAs can avoid degradation and exert biological effects on target cells [18–20]. We found that miR-140-5p was significantly downregulated in exosomes derived from OPLL cells, suggesting that exosomal miR-140-5p may play an important role in OPLL. Here, we explored the mechanism by which exosomal miR-140-5p inhibits osteogenesis in OPLL.

## Results

### MiR-140-5p is significantly downregulated in exosomes derived from OPLL cells

**Fig. 1:**
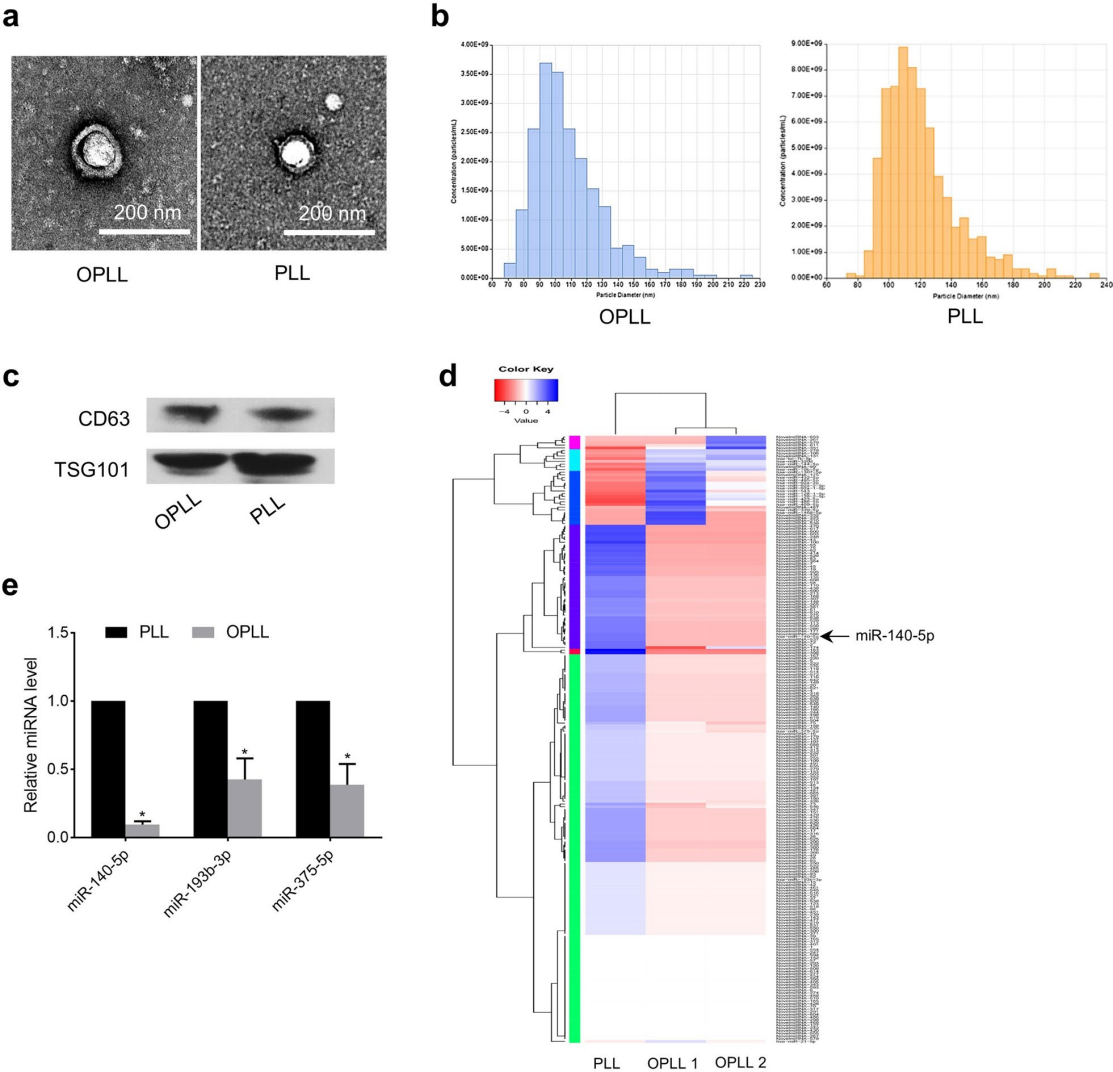
MiR-140-5p is significantly downregulated in exosomes derived from OPLL cells. **a** Representative transmission electron microscopy images of OPLL and PLL cell-derived exosomes. **b** The particle size distribution of collected exosomes by NTA. **c** Western blot of exosomal marker expression in collected exosomes.**d** Heatmap of differentially expressed miRNAs derived from OPLL or PLL exosomes. **e** The downregulation of miR-140-5p was confirmed by qPCR in OPLL tissue. *P < 0.05. All data are presented as the mean ± SD

To clarify the role of exosomes in the pathogenesis of OPLL, we hypothesized that the differentially expressed miRNAs in OPLL cell-derived exosomes played an important role in this process. Therefore, we collected tissue samples from patients with OPLL during surgical operations, cultured cells in vitro using the ligaments close to the ossified mass, and collected exosomes in the culture supernatant of OPLL cells. In addition, we collected the posterior longitudinal ligament (PLL) of patients with cervical spine trauma (non-OPLL) and obtained exosomes from the culture supernatant of PLL cells as a control group. There were no statistic differences between OPLL and non-OPLL patients’ characteristics (Additional file 1: Table S1). The typical double-layer membrane structure of exosomes was observed under transmission electron microscopy (Fig. 1a). Nanoparticle tracking analysis (NTA) showed that the collected extracellular vesicles in each group were approximately 108 nm to 120 nm in size (Fig. 1b). The exosomal markers CD63 and TSG101 (Fig. 1c) were detected. Subsequently, next-generation sequencing (NGS) technology was used to analyze the differentially expressed miRNAs in exosomes (Fig. 1d). We chose the top three downregulated miRNAs for follow-up studies. Next, we used real-time polymerase chain reaction (PCR) to verify NGS data, and the results showed that miR-140-5p was significantly downregulated in OPLL cell-derived exosomes (Fig. 1e).

### Exosomal miR-140-5p is delivered from OPLL cells to hMSCs

**Fig. 2:**
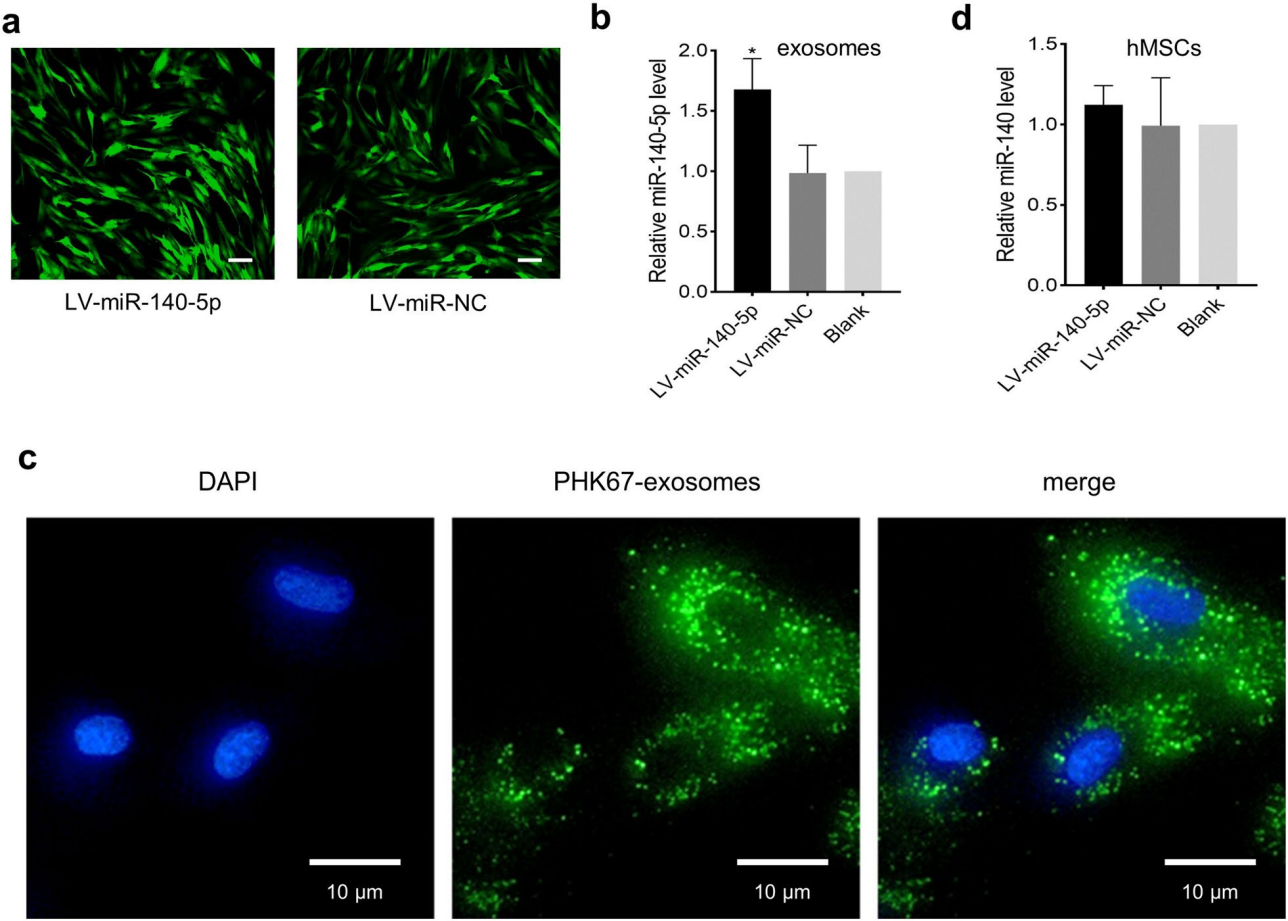
Exosomal miR-140-5p is delivered from OPLL cells into hMSCs. **a** Photos of GFP-positive OPLL cells under fluorescence microscopy indicated that OPLL cells were successfully transinfected with lentivirus. Scale bars = 10 μm. **b** Relative expression of miR-140-5p in OPLL cell exosomes. **c** Delivery of miR-140-5p derived from OPLL cells to hMSCs through exosomes. The green signals were PKH67-labeled exosomes. The cell nuclei were stained blue with DAPI. Scale bars = 10 μm. **d** Relative expression of miR-140 in hMSCs. *P < 0.05. GFP, green fluorescent protein. All data are presented as the mean ± SD

To clarify whether miR-140-5p was secreted by OPLL cells and delivered to hMSCs, OPLL cells were transfected with lentivirus (LV-miR-140-5p) or its negative control to overexpress miR-140-5p (Fig. 2a), and then the cell culture supernatant was collected to isolate exosomes. Quantitative real-time PCR (qPCR) showed that miR-140-5p was enriched in exosomes (Fig. 2b). We labeled miR-140-5p-overexpressing exosomes with PKH67 and then added them to hMSC cultures. PKH67 fluorescence was positive in the cytoplasm of hMSCs (Fig. 2c), indicating that miR-140-5p was delivered by exosomes into hMSCs. Moreover, the expression level of miR-140, the precursor of miR-140-5p, was analyzed in hMSCs cultured with miR-140-5p-overexpressing exosomes or control exosomes (Fig. 2d), showing no significant difference. These results indicated that the upregulation of miR-140-5p in hMSCs was induced by exosomal delivery rather than endogenous miR-140 transcription.

### MiR-140-5p inhibits osteogenic differentiation of hMSCs

**Fig. 3:**
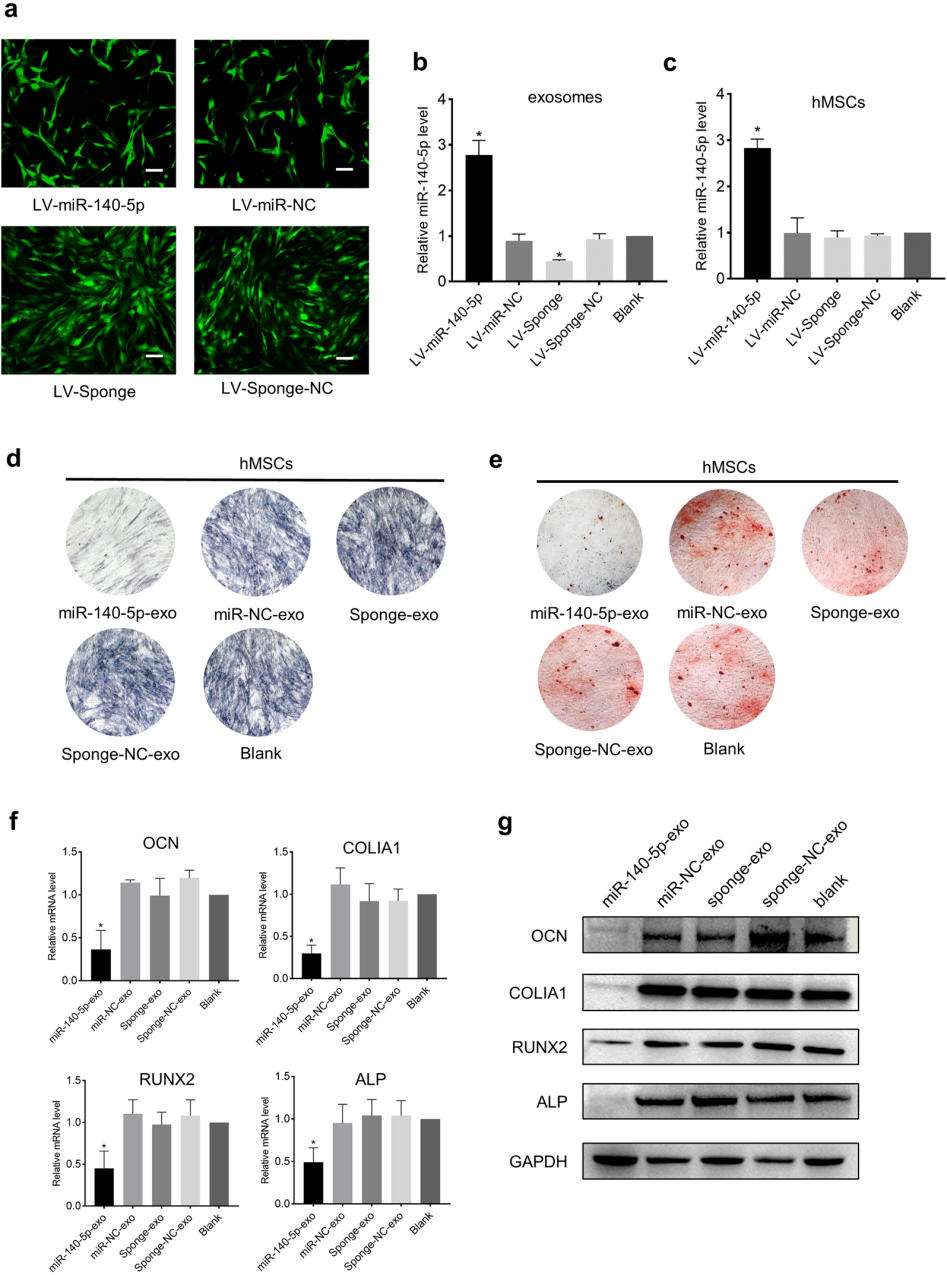
MiR-140-5p inhibits osteogenic differentiation of hMSCs. **a** Photos of GFP-positive OPLL cells under fluorescence microscopy indicated that OPLL cells were successfully transinfected with lentivirus. Scale bars = 10 μm. **b** Relative expression of miR-140-5p in exosomes derived from OPLL cells transfected with LV-miR-140-5p, LV-miR-NC, LV-sponge, or LV-sponge-NC. **c** Relative expression of miR-140-5p in hMSCs, which was delivered by exosomes derived from OPLL cells. The inhibitory effect of miR-140-5p on osteogenesis was confirmed by ALP staining **d** and Alizarin red staining **e**. The relative expression of osteogenesis-related genes was assessed by qPCR **f** and Western blotting **g** All experiments were repeated three times. GAPDH served as the internal control. *P < 0.05. All data are presented as the mean ± SD. LV, lentivirus. NC, negative control. GFP, green fluorescent protein

The prerequisite for the formation of heterotopic ossification is the initiation of osteogenic differentiation of hMSCs. Therefore, to clarify the role of exosomal miR-140-5p in the pathogenesis of OPLL, we studied the effect of exosomal miR-140-5p on the osteogenic differentiation of hMSCs. We transfected OPLL cells (Fig. 3a) with lentivirus LV-miR-140-5p or LV-sponge to overexpress or downregulate miR-140-5p, respectively; the negative control lentivirus LV-miR-NC or LV-sponge-NC was also transfected. We collected cell-conditioned medium to isolate the following exosomes: miR-140-5p-exo, miR-NC-exo, sponge-exo, and sponge-NC-exo. qPCR verified that miR-140-5p was successfully overexpressed or downregulated, as expected, in exosomes (Fig. 3b). After culturing hMSC with the above exosomes for 24 h, qPCR showed that the expression of miR-140-5p in hMSCs was upregulated after miR-140-5p-exo treatment (Fig. 3c), indicating that miR-140-5p was delivered by exosomes into hMSCs. Subsequently, osteogenic induction medium was used to induce osteogenic differentiation of hMSCs. After 7 days, hMSCs were stained with alkaline phosphatase (ALP). After 14 days, hMSCs were stained with Alizarin red. The results showed that the positive rate of hMSC staining after miR-140-5p-exo treatment was significantly lower than that of the other groups (Fig. 3d, e). qPCR and Western blotting showed that the expression of osteogenesis-related genes, including osteocalcin (OCN), collagen type I alpha 1 (COLIA1), runt-related transcription factor 2 (RUNX2), and ALP, in hMSCs treated with miR-140-5p-exo was significantly suppressed (Fig. 3f, g, and Additional file 2: Figure S1a). Taken together, these results indicated that exosomal miR-140-5p inhibited the osteogenic differentiation of hMSCs. The lack of miR-140-5p in exosomes could promote the osteogenic differentiation of hMSCs and the expression of osteogenesis-related genes.

### Insulin-like growth factor 1 (IGF1R) is a direct target of miR-140-5p

**Fig. 4:**
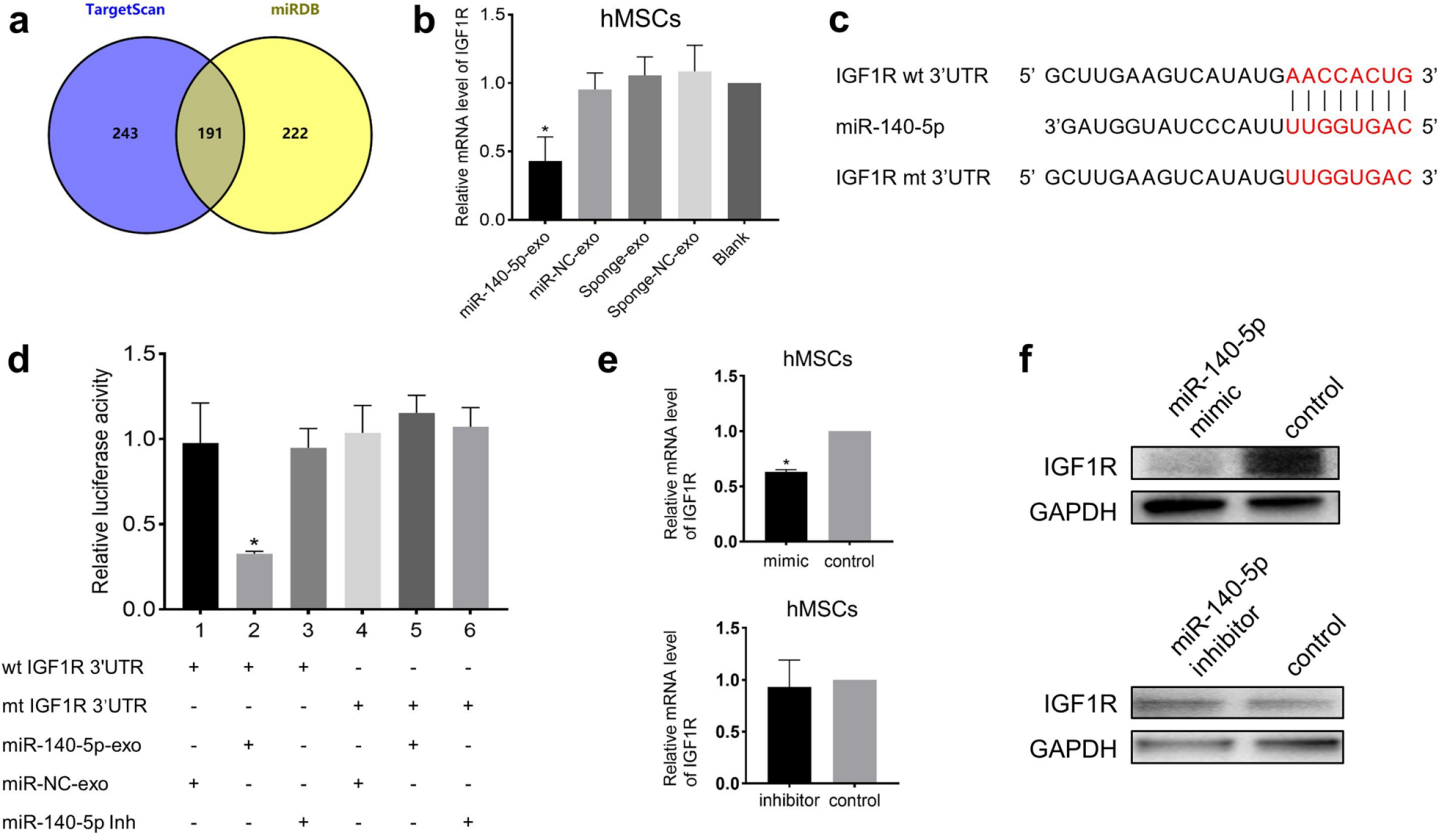
IGF1R is a direct target of miR-140-5p. **a** 191 potential target genes overlapped in the predictions by TargetScan and miRDB. **b** Relative expression of IGF1R assessed by qPCR after treatment with miR-140-5p-exo. **c** Diagram of IGF1R 3’UTR reporter constructs. **d** Luciferase reporter assays in hMSCs transfected with wt or mt IGF1R 3’UTR and exosomes, as indicated. The relative expression of IGF1R after treatment with miR-140-5p mimic or inhibitor was assessed by qPCR **e** and Western blot **f**. All experiments were repeated three times. GAPDH served as an internal control. *P < 0.05. All data are presented as the mean ± SD. wt, wild-type. mt, mutant type

To clarify the mechanism by which miR-140-5p inhibits the osteogenic differentiation of hMSCs, we used the TargetScan database to predict target genes of miR-140-5p, and 434 transcripts were found. We also used the miRDB database to predict the targets, and there were 413 predicted targets for miR-140-5p in miRDB. To narrow down the possible candidate genes, we determined the overlap of the above two databases, which showed 191 genes in total (Fig. 4a). One of them was IGF1R, which belongs to the receptor tyrosine kinase family. The insulin receptor subfamily is located on the cell membrane and can be activated by insulin-like growth factors (IGF1 and IGF2) to cause phosphorylation of its own tyrosine kinase domain, initiates intracellular signal transduction, and regulates cell growth, differentiation, and various life activities, such as the growth, development, and aging of organisms [21, 22]. We tested the mRNA level of IGF1R in hMSCs after miR-140-5p-exo treatment, and the results showed that the mRNA level of IGF1R was significantly reduced (Fig. 4b). We used a luciferase reporter assay to explore whether IGF1R was the direct target of miR-140-5p. The target sequences of IGF1R (wt 3’UTR and mt 3’UTR) were cloned into a vector (Fig. 4c). Subsequently, hMSCs were transfected with the vector and cocultured with miR-140-5p-exo. The results showed that the fluorescence intensity in the wt 3'UTR carrier was significantly reduced, while the fluorescence intensity in the mt 3'UTR carrier was not significantly different from that in the control group (Fig. 4d). In addition, we also directly transfected miR-140-5p mimic or inhibitor into hMSCs, and the results showed that IGF1R mRNA and IGF1R protein were downregulated by miR-140-5p mimic (Fig. 4e, f, and Additional file 2: Figure S1b). Taken together, these results indicated that IGF1R was a direct target of miR-140-5p. Exosomal miR-140-5p interacted with the IGF1R 3'UTR and exerted a transcriptional inhibitory effect in hMSCs.

### Exosomal miR-140-5p derived from OPLL cells targets IGF1R and regulates the mTOR pathway

**Fig. 5:**
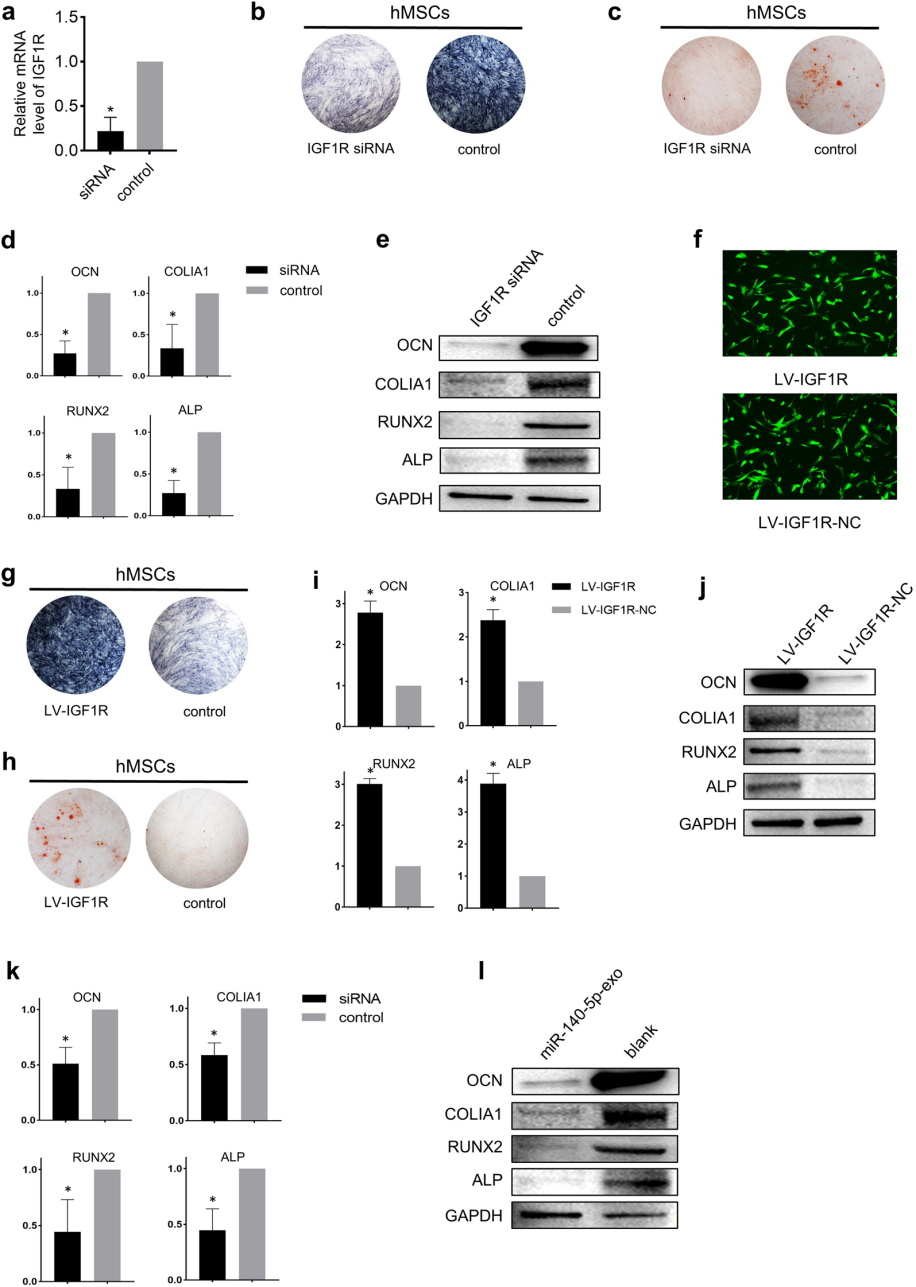
IGF1R is essential for exosomal miR-140-5p to inhibit osteogenesis of hMSCs. **a** Relative expression of IGF1R assessed by qPCR after treatment with IGF1R siRNA. Osteogenesis after treatment with IGF1R siRNA confirmed by ALP staining **b** and Alizarin red staining **c**. The relative expression of osteogenesis-related genes was assessed by qPCR **d** and Western blotting **e**, **f** Photos of GFP-positive hMSCs under fluorescence microscopy indicated successful transfection. Scale bars = 10 μm. Osteogenesis after transfection with LV-IGF1R was confirmed by ALP staining **g** and Alizarin red staining **h**. The relative expression of osteogenesis-related genes was assessed by qPCR **i** and Western blotting **j**. After transfection with LV-IGF1R, miR-140-5p-exo reversed the osteogenic effect of IGF1R, and the relative expression of osteogenesis-related genes was assessed by qPCR **k** and Western blotting **l** All experiments were repeated three times. GAPDH served as an internal control. *P < 0.05. All data are presented as the mean ± SD. LV, lentivirus. NC, negative control. GFP, green fluorescent protein

To clarify that the inhibitory effect of exosomal miR-140-5p on the osteogenic differentiation of hMSCs was mediated by the inhibition of IGF1R, we used IGF1R siRNA to directly knock down IGF1R in hMSCs (Fig. 5a). After osteogenic induction of the hMSCs, the positive rate of ALP and Alizarin Red staining of hMSCs in the knockdown group was significantly lower than that of the control group (Fig. 5b, c). The expression of OCN, COLIA1, RUNX2, and ALP was also suppressed (Fig. 5d, e, and Additional file 2: Figure S1c). We also performed an IGF1R rescue assay. LV-IGF1R was transfected into hMSCs to overexpress IGF1R (see Fig. 5f for transfection efficiency), and osteoinduction medium was used for differentiation. The results showed that after IGF1R was upregulated, ALP and Alizarin Red staining were strongly positive (Fig. 5g, h). The expression levels of OCN, COLIA1, RUNX2, and ALP also increased (Fig. 5i, j, and Additional file 2: Figure S1d). Moreover, after hMSCs were transfected with LV-IGF1R, we treated them with miR-140-5p-exo, and the effect of IGF1R on promoting osteogenesis was reversed (Fig. 5k, l, and Additional file 2: Figure S1e).

**Fig. 6:**
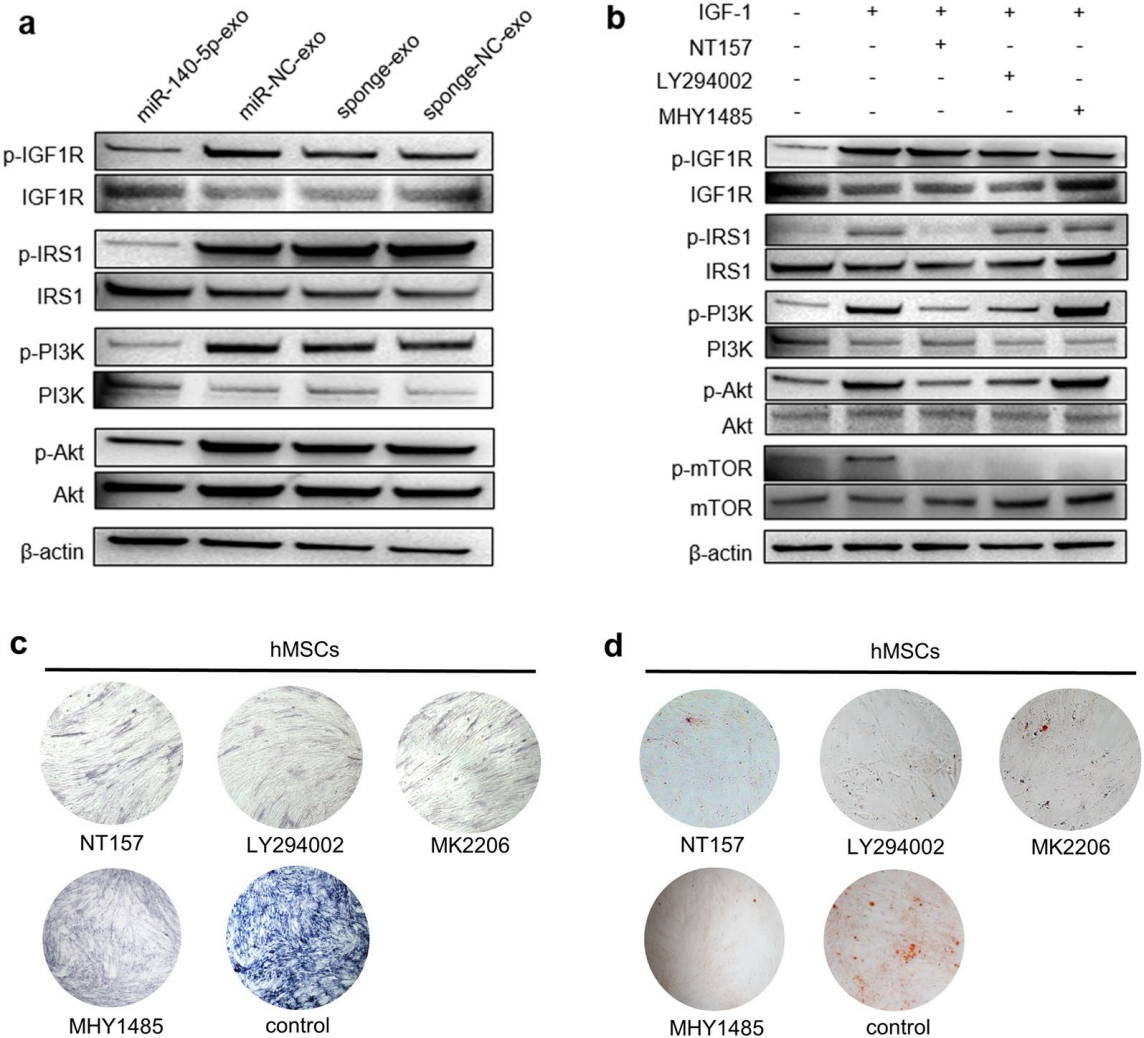
Exosomal miR-140-5p inhibits osteogenesis by regulating the mTOR pathway through the IGF1R/IRS1/PI3K/Akt axis. **a** Western blot analysis of IGF-1-induced phosphorylation of IGF1R, IRS1, PI3K, Akt, and mTOR in hMSCs treated with miR-140-5p-exo, miR-NC-exo, sponge-exo, or sponge-NC-exo. **b** Western blot analysis of IGF-1-induced phosphorylation of IGF1R, IRS1, PI3K, Akt, and mTOR in hMSCs treated with or without NT157 (1.6 μM), LY294002 (10 μM), or MHY1485 (20 nM) for 20 min. Osteogenesis after treated with NT157, LY294002, MK2206, or MHY1485 was confirmed by ALP staining **c** and Alizarin red staining **d**. All experiments were repeated three times. β-actin served as an internal control. *P < 0.05. All data are presented as the mean ± SD

To explore the molecular mechanism of exosomal miR-140-5p inhibiting osteogenesis through IGF1R, we mainly examined the mTOR pathway related to the osteogenic differentiation of hMSCs because it has been reported in the literature that the combination of IGF1 and IGF1R can promote the osteogenesis of MSCs by activating the mTOR pathway, resulting in improvement of osteoporosis in mice [23]. After we treated hMSCs with miR-140-5p-exo, IGF1 did not stimulate the phosphorylation of IGF1R, IRS1, PI3K, Akt, or mTOR, while after miR-NC-exo, sponge-exo, or sponge-NC-exo treatment, IGF1 stimulated the phosphorylation of IGF1R, IRS1, PI3K, Akt, and mTOR (Fig. 6a and Additional file 3: Figure S2a). To clarify the upstream and downstream relationships of IRS1, PI3K, Akt, and mTOR, we treated hMSCs with the IRS1 inhibitor NT157. The results showed that IGF1 could not cause the phosphorylation of PI3K, Akt, or mTOR. LY294002 (PI3K inhibitor) treatment reduced the phosphorylation of Akt and mTOR caused by IGF1 but did not affect the phosphorylation of IGF1R and IRS1. In addition, MHY1485 (mTOR inhibitor) treatment did not inhibit the phosphorylation of IRS1, PI3K, or Akt caused by IGF1 (Fig. 6b and Additional file 3: Figure S2b). Therefore, the upstream and downstream relationship of the above key molecules was IGF1R/IRS1/PI3K/Akt/mTOR. To further clarify the role of the IGF1R/IRS1/PI3K/Akt/mTOR axis, we respectively used NT157 (IRS1 inhibitor), LY294002 (PI3K inhibitor), MK2206 (Akt inhibitor), or MHY1485 (mTOR inhibitor) to interfere with the osteogenic induction of hMSCs. The results showed that NT157, LY294002, MK2206, or MHY1485 inhibited the osteogenic differentiation of hMSCs, respectively (Fig. 6c, d). Taken together, these data indicated that exosomal miR-140-5p inhibited the osteogenic differentiation of hMSCs by targeting IGF1R and regulating the mTOR pathway.

### MiR-140-5p inhibits bone formation in vivo

**Fig. 7:**
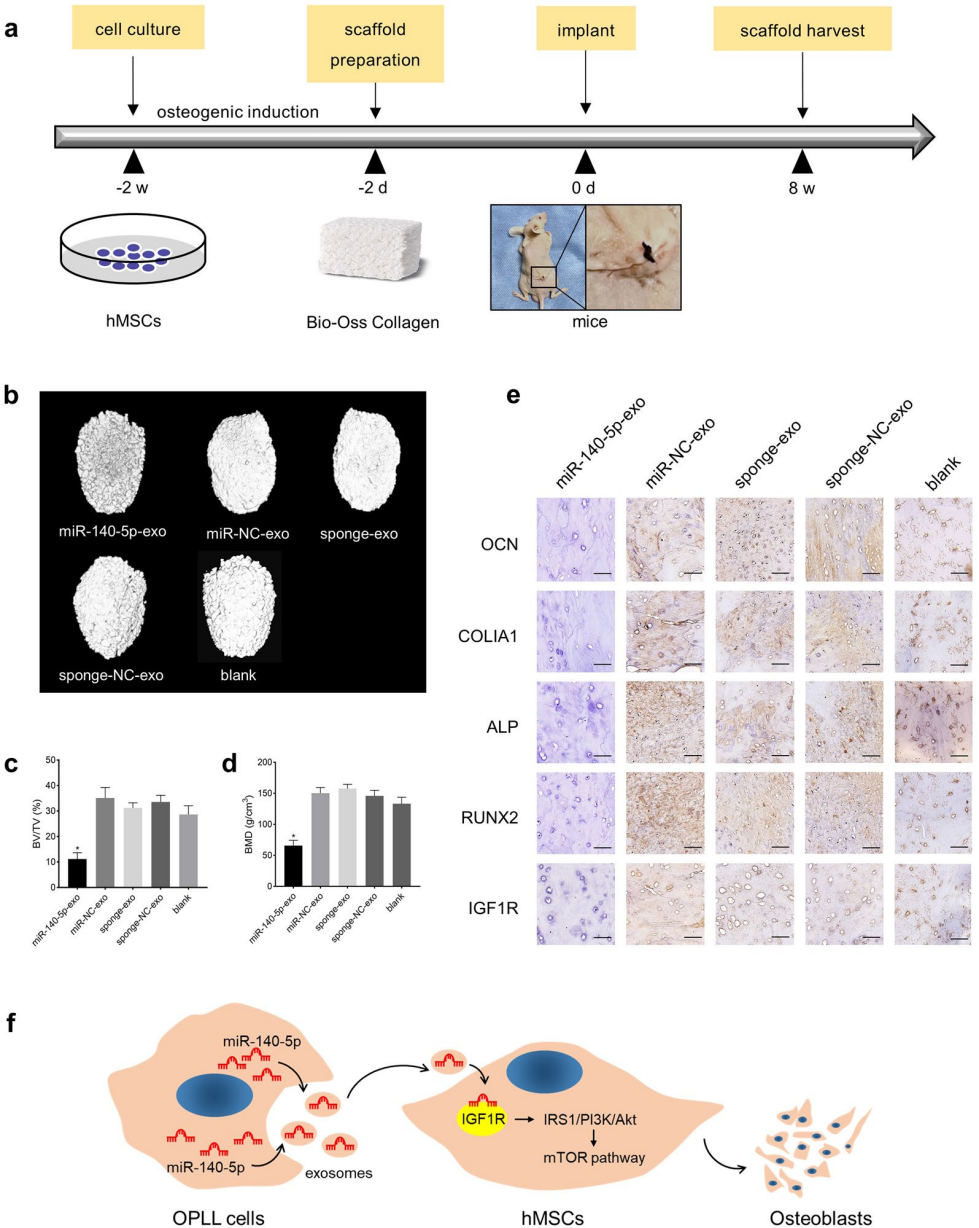
MiR-140-5p inhibits bone formation in vivo. **a** Diagram of the procedures of the bone formation experiment in vivo*.* **b** 3D-reconstructed images of scaffolds after 8 weeks. BV/TV **c** and BMD **d** of scaffolds were calculated by micro-CT scan among different groups treated with miR-140-5p-exo, miR-NC-exo, sponge-exo, or sponge-NC-exo. **e** Immunohistochemical staining of scaffolds. Scale bars = 10 μm. **f** Schematic diagram of the mechanism by which exosomal miR-140-5p from OPLL cells inhibited osteogenesis by targeting IGF1R and regulating the mTOR pathway. For each group, n = 5. *P < 0.05. All data are presented as the mean ± SD

To further explore the function of miR-140-5p in vivo, we conducted ectopic bone formation experiments in nude mice. We cultured hMSCs with miR-140-5p-exo, miR-NC-exo, sponge-exo, or sponge-NC-exo for 48 h. After osteogenic induction, the above hMSCs and Bio-Oss Collagen were thoroughly mixed and cultured for 48 h. Finally, the mixture of scaffold and cells was implanted under the skin of the backs of nude mice (Fig. 7a). The animals were sacrificed 8 weeks later, and the bone volume/tissue volume (BV/TV) and bone mineral density (BMD) were calculated by micro computed tomography (micro-CT) scans (Fig. 7b). The results showed that after miR-140-5p-exo treatment, the BV/TV and BMD of the ectopic bone significantly decreased (Fig. 7c and d). Subsequently, we performed immunohistochemical detection of ectopic bone, and the results showed that the expression of OCN, COLIA1, RUNX2, and ALP in ectopic bone was inhibited after miR-140-5p-exo treatment, while the control groups were positive (Fig. 7e). In addition, the ectopic bone sections were also immunostained by IGF1R, and the results showed that the positive rate of IGF1R in the ectopic bone treated with miR-140-5p-exo was significantly lower than those of the control groups (Fig. 7e). The above results indicated that miR-140-5p had an inhibitory effect on bone formation in vivo.

In summary, our study found that the expression of exosomal miR-140-5p in OPLL cells was significantly lower than that in PLL cells. MiR-140-5p was transferred into hMSCs by exosomes, targeted IGF1R, regulated IRS1/PI3K/Akt, and ultimately inhibited osteogenic differentiation through the mTOR pathway. Overexpression of miR-140-5p in OPLL cells inhibited the osteogenic differentiation of hMSCs, which is a potential new strategy for OPLL treatment. A schematic diagram of this mechanism is shown in Fig. 7f.

## Discussion

OPLL is a common disease requiring spinal surgery and is one of the causes of cervical spinal cord compression and paralysis. It is highly prevalent in East Asian populations [2, 3]. Due to insufficient understanding of the mechanism of spinal ligament ossification, there are currently no effective nonsurgical methods to prevent or cure OPLL. Patients often experience poor surgical outcomes, including ossified mass growth and recurrence or even aggravation of symptoms after surgery [24]. Therefore, the development of nonsurgical treatments for early intervention of OPLL is particularly important, especially exploring the key molecules and signaling pathways in the pathogenesis of OPLL for possible therapeutic targets. It was reported in the literature that the occurrence of OPLL may be related to multiple factors, such as heredity, environment, and lifestyle. However, how these factors induce ectopic osteogenesis through specific molecular mechanisms has not yet been elucidated [25, 26]. At present, it is generally accepted that multiple factors promote the osteogenic differentiation of MSCs in the posterior longitudinal ligaments, thereby initiating heterotopic ossification. Although studies have shown that MSCs exist in spinal ligaments (e.g., ligamentum flavum, posterior longitudinal ligament, and interspinous ligament) [8, 27], the specific osteogenic differentiation mechanism has not been elucidated in detail.

The extracellular microenvironment plays an important role in cell proliferation, differentiation, metabolism, and other biological activities, and changes in microenvironmental components may lead to abnormal cell differentiation [28, 29]. Almost all somatic cells can secrete microvesicles into the extracellular microenvironment; extracellular microvesicles with a diameter of 50–150 nm are called exosomes [14]. Exosomes contain DNA, mRNA, noncoding RNA (including miRNA, lncRNA, circRNA, etc.), lipids and proteins. These biologically active substances participate in the regulation of a variety of physiological activities of recipient cells [14]. Exosomal miRNA participates extensively in the regulation of cell physiological activities after entering recipient cells and may play a role in promoting the osteogenic differentiation of MSCs. According to the literature, miR-193b-3p can regulate chondrogenesis and the metabolic activity of chondrocytes; exosomal miR-320c can promote the differentiation of bone marrow MSCs to chondrocytes; and osteoclast exosomal miR-214-3p can inhibit the bone formation process of osteoblasts [30–32]. These studies all suggested that exosomal miRNA plays an important regulatory role in the process of bone formation after entering the recipient cells. We isolated the exosomes secreted by OPLL cells, analyzed the differentially expressed miRNAs, and found that the expression of miR-140-5p decreased significantly. We constructed miR-140-5p-overexpressing exosomes and labeled the exosomes with PKH67. We proved that exosomes could deliver miR-140-5p into hMSCs. In addition, we demonstrated through a series of functional experiments that miR-140-5p could inhibit osteogenic differentiation after being delivered by exosomes into hMSCs. Therefore, we speculate that the lack of miR-140-5p relatively weakened the inhibition of osteogenic differentiation and finally initiated the osteogenic process.

IGF-1 binds to IGF1R to activate it, resulting in the regulation of cell growth and differentiation and promoting the mineralization process coupled with osteoblasts, thereby promoting bone formation [33, 34]. The activation of IGF1R can activate autophagy, which is a joint process that stimulates the differentiation of early osteoblasts [35]. Goto et al. [36] found that IGF-1 was highly expressed in OPLL tissue sections and induced osteogenic differentiation. Therefore, IGF-1 might be involved in the ossification process of OPLL. Similar to these studies, we found that IGF1R was one of the candidate targets when predicting miR-140-5p target genes. Subsequently, we used a luciferase reporter assay to demonstrate the interaction between miR-140-5p and IGF1R. In addition, we used LV-IGF1R or siRNA to treat hMSCs to overexpress or downregulate IGF1R in hMSCs, respectively. It was found that the osteogenic differentiation of hMSCs was promoted or inhibited as expected, proving the role of IGF1R in regulating osteogenesis. We further clarified that IGF1R promoted the phosphorylation of IRS1/PI3K/Akt and activated the mTOR pathway to promote osteogenesis. In one study, Xian et al. [23] found that IGF-1 improved osteoporosis through the mTOR pathway, which also supported our results. In our study, overexpression of miR-140-5p exosomes reversed the promotion in osteogenesis caused by the upregulation of IGF1R and inhibited the osteogenic differentiation of hMSCs, suggesting that drugs targeting IGF1R on the surface of MSCs in the posterior longitudinal ligament (such as exosomes delivering miR-140-5p) may serve as a therapy for OPLL in the future.

There are some limitations in this study. OPLL is considered to be a polygenic genetic disease, and multiple factors costimulate the expression of susceptible genes, which leads to the initiation of heterotopic ossification. However, the content of the extracellular microenvironment is extremely rich, and the role of extracellular vesicles represented by exosomes in the pathogenesis of OPLL may have limitations. In addition, exosomes carry a large number of biologically active molecules, including miRNA, mRNA, and proteins. Therefore, the role of exosomal miR-140-5p in the extracellular microenvironment needs to be further investigated. The role of exosomal miR-140-5p in vivo was examined in nude mice with a Bio-Oss Collagen scaffold. The reason is that there is no reliable animal model of OPLL. In addition to the ossification of the spinal ligament, the tip-toe walking mice commonly used by scholars show ossification and mineralization of multiple tissues and organs throughout the body, including the thoracic aorta, carotid artery, heart, spleen, lung, eye, kidney, and liver [37, 38]. Thus, this mouse model cannot completely simulate the pathological process of OPLL in the human body because OPLL is an isolated disease, usually not accompanied by ossification of other tissues and organs.

Taken together, this study demonstrated that exosomal miR-140-5p inhibited the osteogenic differentiation of hMSCs by targeting IGF1R and regulating the mTOR pathway and played an important role in the pathogenesis of OPLL. This research focused on biologically active molecules in the extracellular microenvironment of OPLL for the first time and clarified the role and mechanism of hMSCs in the formation of OPLL, which could provide a new theoretical basis for the future development of targeted drugs for the treatment of OPLL.

## Conclusions

Our data indicated that exosomal miR-140-5p could inhibit the osteogenic differentiation of hMSCs by targeting IGF1R and regulating the mTOR pathway. The findings in this study indicated that exosomes, as a drug delivery system, might serve as a potential means of drug treatment for OPLL treatment in the future. IGF1B might also be a target for future molecular therapy of OPLL.

## Methods

### Clinical sample collection, cell culture, and osteogenic induction

A total of 20 OPLL patient samples and 18 cervical spine trauma patients' PLL samples were collected. The characteristics of patients were displayed in Additional file 1: Table S1. All experiments in this study were approved by the ethics committee of Shanghai Changzheng Hospital. The patients signed an informed consent form before the operation. The OPLL samples came from patients who had received anterior cervical corpectomy and fusion (ACCF). The inclusion criteria were patients [1] who were diagnosed with cervical OPLL or cervical spine trauma through X-ray, MRI and CT; [2] who received ACCF. The exclusion criteria were patients with [1] a history of infection, tumor, osteoporosis or other serious neurological diseases; [3] a history of substance abuse.

After the vertebral body was removed during the operation, the ossified posterior longitudinal ligament of the posterior wall of the vertebral body was carefully separated, washed with normal saline, and stored aseptically in complete cell culture medium, which was prepared for primary OPLL cell culture. The normal posterior longitudinal ligament was derived from patients with cervical spine trauma who received ACCF. The posterior longitudinal ligament behind the posterior wall of the vertebral body was also separated for primary PLL cell culture. Specifically, tissues from patients with OPLL or PLL were cut into pieces with a diameter of approximately 1 mm and placed in a petri dish with complete cell culture medium (90% high-glucose Dulbecco’s modified Eagle’s medium (DMEM, Gibco, USA), 10% fetal bovine serum (FBS, Gibco, USA), and 1% penicillin/streptomycin (Gibco, USA)). The petri dishes were placed into a 37 °C constant temperature incubator in a humidified atmosphere containing 5% CO_2_. Half of the medium was changed every 3 days, and media was completely changed every 7 days. After 10–14 days, a mass of fibroblast-like cells migrated out of the tissue and grew adherently, which can be observed microscopically. When the cell confluence reached 80%, the cells were passaged at a ratio of 1:3. According to literature, cells derived from OPLL mainly consist of fibroblasts, osteoblasts, and chondrocytes [39, 40]. In this article, we define these cells as OPLL cells.

The hMSCs used in this experiment were purchased from the Cell Bank of the Chinese Academy of Sciences (Shanghai, China). The complete cell culture medium consisted of 90% low-glucose DMEM (Gibco, USA), 10% FBS (Gibco, USA), and 1% penicillin/streptomycin (Gibco, USA). The culture environment was the same as that of OPLL cells.

### Isolation and identification of exosomes

The exosomes were separated from the cell culture medium with gradient centrifugation. The OPLL cell or PLL cell supernatant was centrifuged at 300 × g for 15 min to remove floating cells and then centrifuged again at 820 × g for 15 min and 10,000 × g for 5 min, and cell debris was removed through a 0.8 μm filter. Finally, the samples were centrifuged at 100,000 × g for 2 h (Beckman L-90 K, USA) to obtain an exosome pellet. The exosomal pellet was resuspended in PBS and ultracentrifuged again for further experiments. All centrifugation was performed at 4 °C.

The size distribution and concentration of exosomes were examined by nanoparticle tracking analysis with ZetaView PMX 110 (Particle Metrix, Germany) and the corresponding software ZetaView 8.04.02. Exosomes were diluted in PBS to measure the particle size and concentration. NTA measurements were recorded and analyzed at 11 positions. The ZetaView system was calibrated using 110 nm polystyrene particles. All procedures were performed at room temperature. The exosome samples were prepared and observed with a transmission electron microscope (H-7650, Hitachi, Japan) as described previously [41]. The voltage was 80 kV.

### Analysis of differentially expressed miRNAs and target prediction

Total exosomal RNA of OPLL or PLL cells was extracted using a Total Exosome RNA Isolation Kit (Invitrogen, USA) according to the manufacturer’s instructions. MiRNA library construction and miRNA sequencing were performed as described previously [42] to identify the differentially expressed miRNAs derived from OPLL or PLL cell exosomes. Next, target genes of miR-140-5p were predicted by TargetScan (http://www.targetscan.org) and miRDB (http://mirdb.org). The intersection of these two databases was created by Venny 2.1.0 (https://bioinfogp.cnb.csic.es/tools/venny).

### Exosome labeling and confocal microscopy

Exosomes were collected and labeled with the green lipophilic fluorescent dye PKH67 (Sigma, USA) and then cocultured with hMSCs for 1 h. After that, hMSCs were washed with PBS and fixed with 4% paraformaldehyde. The cell nuclei were stained with 4′6-diamidinophenylidole (DAPI, Beyotime, China). The samples were observed with confocal microscopy (FV10i, Olympus, Japan).

### Luciferase reporter assay

With the help of restriction endonuclease site primers, we synthesized a human wild-type IGF1R 3'UTR fragment containing the miR-140-5p conserved binding site by PCR and cloned it into the pMIR reporter vector. This is called the wild-type (wt) IGF1R 3'UTR. By mutating the miR-140-5p binding site of the wt IGF1R 3'UTR, a mutant (mt) IGF1R 3'UTR was synthesized and cloned into the vector. Human 293 T cells were transfected with the above two vectors and cultured with miR-140-5p-exo or control conditions. A dual luciferase reporter gene detection system (Promega, USA) was used to detect luciferase activity.

### Transfection of lentivirus and oligonucleotide

Agomirs, antagomirs, and siRNAs were synthesized by Sangon Biotech (Shanghai, China). The IGF1R vector for cDNA delivery was synthesized by Sangon Biotech (Shanghai, China). Lentivirus production, purification, and titration were performed as described previously [43]. Transfection of miR-140-5p mimic or inhibitor into cells was performed by using Lipofectamine 2000 (Invitrogen, USA) according to the manufacturer’s instructions. Transfection of miR-140-5p mimic or inhibitor into exosomes was performed by using Exo-Fect Exosome Transfection Kit (SBI, CA) according to the manufacturer’s instructions.

### In vivo Bio-Oss collagen scaffold implantation and bone formation

After miR-140-5p-exo, miR-NC-exo, sponge-exo, and sponge-NC-exo were collected, they were added to hMSC culture for 48 h and then replaced by osteogenic induction culture for 14 days. The cells were resuspended, thoroughly mixed with Bio-Oss collagen (Geistlich, GEWO GmbH, Germany) and cultured for 48 h. Finally, the mixture of scaffold and cells was implanted into the backs of nude mice (4-week-old BALB/c homozygous nude) subcutaneously. After antiseptic preparation of the operative site, a 2 mm-long incision was made. The incision was located on the back of the mouse to avoid possible bites from itself. The incision was normally 0.5 mm deep, which was enough to reach the surface of the muscle. The space superior to the muscle was bluntly dissected. Then the prepared scaffold was implanted into the space and the skin was sutured. After 8 weeks, the specimen was removed and fixed in 4% paraformaldehyde for further immunohistochemical analysis. Images of animal models after 8 weeks were shown in Additional file 4: Figure S3.

### Immunohistochemical analysis

The scaffold samples were decalcified in 10% ethylene diamine tetraacetic acid (EDTA) for 1 month. Then, they were dehydrated and embedded in paraffin. The paraffin tissue block was cut into 5 μm slices for hematoxylin and eosin (H&E) staining. The sections were blocked with 3% BSA for 30 min and then incubated with primary antibodies (Abcam, USA), including anti-OCN, anti-COLIA1, anti-RUNX2 anti-ALP, and anti-TGF1R. Then, samples were placed at 95 °C in citrate buffer to retrieve antigens for 10 min. Subsequent sections were incubated again with primary antibodies at 4 °C overnight. Sections were observed with an Olympus microscope equipped with an Olympus DP70 camera (Olympus, Japan).

### Micro-CT scan

The scaffold samples were harvested after intervention and fixed in 4% paraformaldehyde. Subsequently, the samples were scanned by micro-CT (Quantum FX microCT, PerkinElmer, USA); RigakuTM software (Rigaku, Japan) and RadiAnt DICOM ViewerTM (Medixant, Poland) were used for 3D reconstruction and image processing.

### ALP and alizarin red staining

Osteogenesis induction medium (Cyagen, China) contained 10% FBS, 1% penicillin–streptomycin, 10 mM L-glutamine, 50 μM L-ascorbic acid, 10 mM β-glycerophosphate, and 100 nM dexamethasone. When the degree of hMSC fusion reached 60%-70%, the culture medium was replaced with osteogenic medium for osteoinduction. The medium was changed every 3 days. After 14 days, the induction medium was removed. Then, the cells were washed with PBS and fixed with 4% paraformaldehyde. Cells were stained by using an ALP staining kit (Beyotime, China) according to the manufacturer’s instructions. Similarly, cells were stained with Alizarin red S (Cyagen, China) 14 days after induction.

### RNA extraction and qPCR

Briefly, total RNA from cultured cells was isolated by TRIzol (Invitrogen, USA) according to the manufacturer's instructions. Then, the extracted RNA was reverse transcribed into cDNA by a ReverTra Ace® qPCR RT Kit (Toyobo, Japan). qPCR was performed with real-time PCR (ABI 7500, Applied Biosystems, USA), and the expression levels of several osteogenesis-related genes, including OCN, COLIA1, RUNX2 and ALP, were calculated by the 2^−ΔΔCt^ method. The sequences of qPCR primers were presented in Additional file 5: Table S2.

### Western blot

First, cells were lysed with high-efficiency RIPA lysis buffer (Solarbio, China), and the protein content was determined by a BCA protein assay kit (Thermo Fisher Scientific, USA). Second, protein samples were separated by Bis–Tris gel (Invitrogen, USA), transferred to a nitrocellulose filter membrane (Bio-Rad, USA), blocked with 5% w/v skimmed milk in TBST buffer (1X Tris-buffered saline and 0.1% Tween 20) for 1 h at room temperature, and incubated with primary antibodies overnight at 4 °C. Third, the membranes were washed and incubated with secondary antibodies for 2 h at room temperature. Finally, the Odyssey imaging system (Li-Cor, Lincoln, USA) was used to determine the fluorescent signals. Anti-GAPDH or anti-β-actin was used as an endogenous control.

### Statistical analysis

All data analysis was performed using SPSS 21.0 software (Chicago, IL, USA). All data are expressed as the mean ± SD. Student’s t test was selected for the comparison of two independent variables. One-way ANOVA was selected for the comparison of multiple independent variables. A P value < 0.05 was considered to be statistically significant.

## Supplementary Information

**Additional file 1: Table S1.** Characteristics of patients.

**Additional file 2. Figure S1. **Quantitative analysis of the osteogenesis-related proteins.

**Additional file 3. Figure S2. **Quantitative analysis of the phosphorylation of the proteins.

**Additional file 4. Figure S3. **Representative photos of the animal model.

**Additional file 5: Table S2.** The sequences of qPCR primers.

## Data Availability

The raw data used and/or analyzed in this research are available from the corresponding author upon reasonable request.
