# Supplementary material for: Exosomal miR-140-5p inhibits osteogenesis by targeting IGF1R and regulating the mTOR pathway in ossification of the posterior longitudinal ligament
Source: J Nanobiotechnology. 2022 Oct 15;20:452. doi: 10.1186/s12951-022-01655-8 (PMC9571456; doi:10.1186/s12951-022-01655-8)
Supplement: Supplementary file 1 — Additional file 1: Table S1. Characteristics of patients. [file 12951_2022_1655_MOESM1_ESM.docx]

**Supplementary Table S1. The sequences of qPCR primers.**

| Primer Name | Sequence（5′-3′） |
| --- | --- |
| miR-140-5p | RT: CTCAACTGGTGTCGTGGAGTCGGCAATTCAGTTGAGCTACCAT |
|  | F: ACACTCCAGCTGGGCAGTGGTTTTACCCT |
| OCN | F: AGGGCAGCGAGGTAGTGAAGAG |
|  | R: GGTCAGCCAACTCGTCACAGTC |
| COLIA1 | F: TGATCGTGGTGAGACTGGTCCTG |
|  | R: CTTTATGCCTCTGTCGCCCTGTTC |
| RUNX2 | F: AGGCAGTTCCCAAGCATTTCATCC |
|  | R: TGGCAGGTAGGTGTGGTAGTGAG |
| ALP | F: GCCTACACGGTCCTCCTATACGG |
|  | R: CACTGCTGACTGCTGCCGATAC |
| IGF1R | F: TGCTGACCTCTGTTACCTCTCCAC |
|  | R: GTCTTCTCACACATCGGCTTCTCC |
| GAPDH | F: ATCCCATCACCATCTTCC |
|  | R: GAGTCCTTCCACGATACCA |
| β-actin | F: CTCCATCCTGGCCTCGCTGT |
|  | R: GCTGTCACCTTCACCGTTCC |
