# Supplementary figures and images for: Exosomal miR-140-5p inhibits osteogenesis by targeting IGF1R and regulating the mTOR pathway in ossification of the posterior longitudinal ligament
Source: J Nanobiotechnology. 2022 Oct 15;20:452. doi: 10.1186/s12951-022-01655-8 (PMC9571456; doi:10.1186/s12951-022-01655-8)

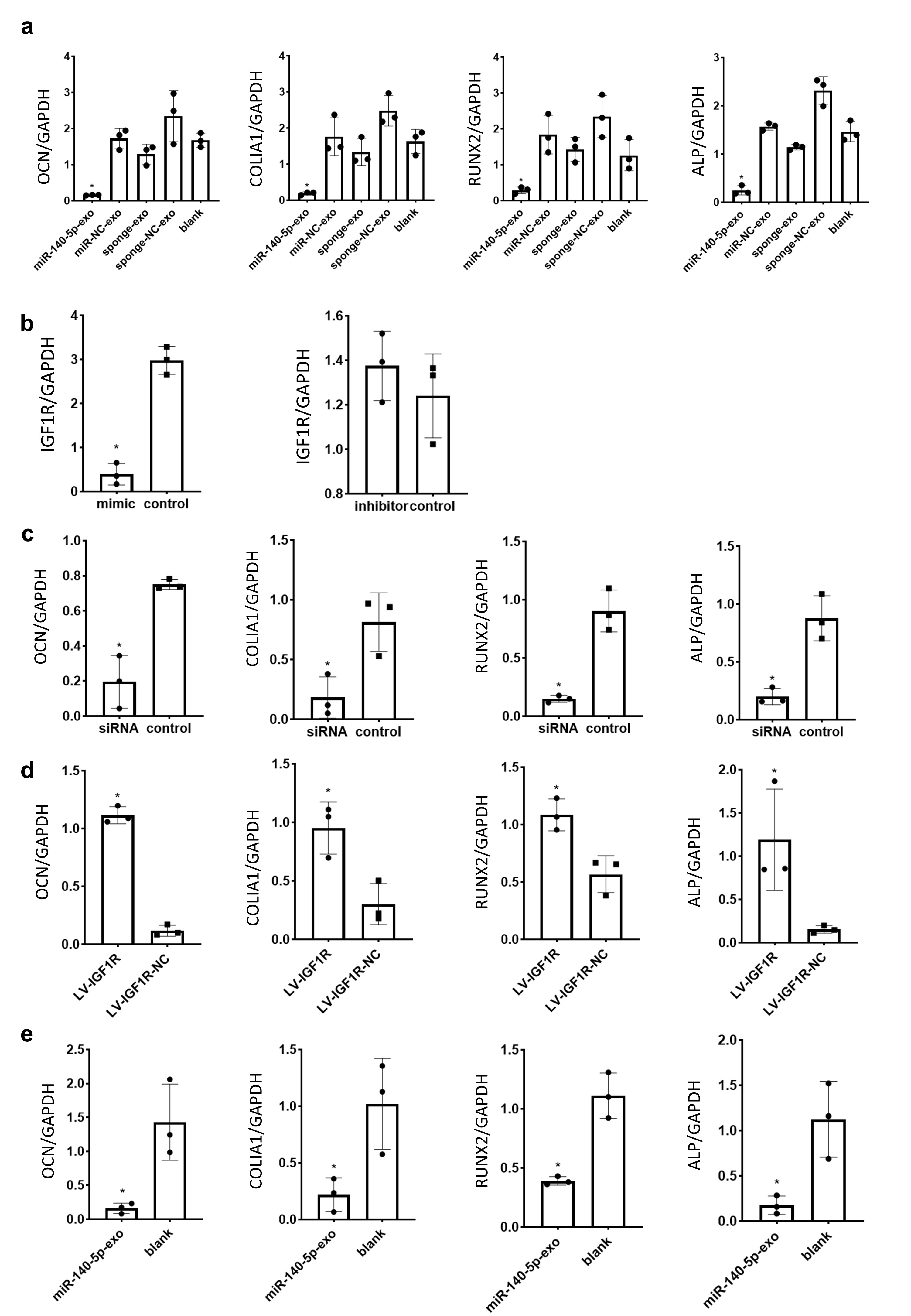

Supplement: Supplementary file 2 — Additional file 2. Figure S1. Quantitative analysis of the osteogenesis-related proteins. [file 12951_2022_1655_MOESM2_ESM.jpg]

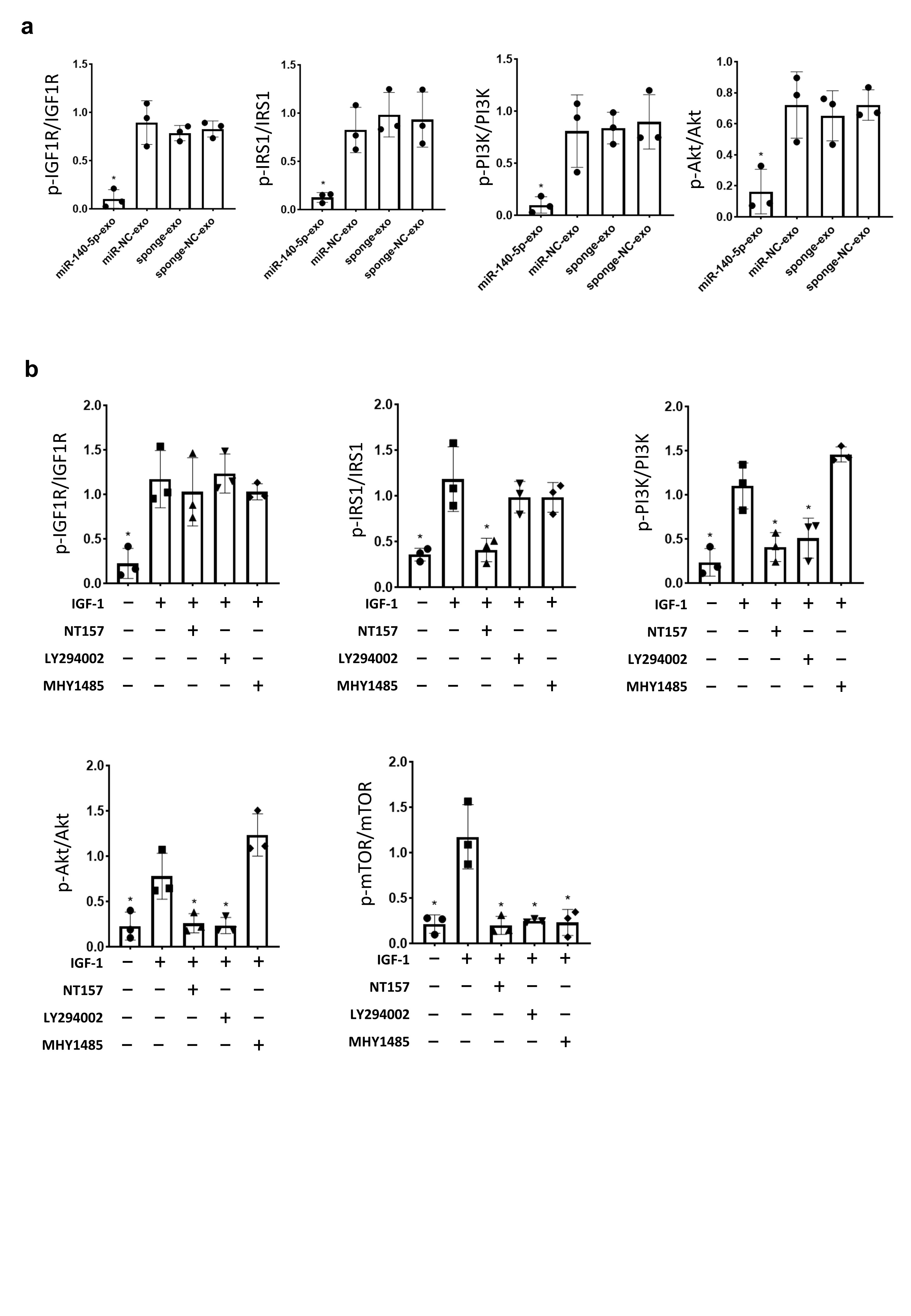

Supplement: Supplementary file 3 — Additional file 3. Figure S2. Quantitative analysis of the phosphorylation of the proteins. [file 12951_2022_1655_MOESM3_ESM.jpg]

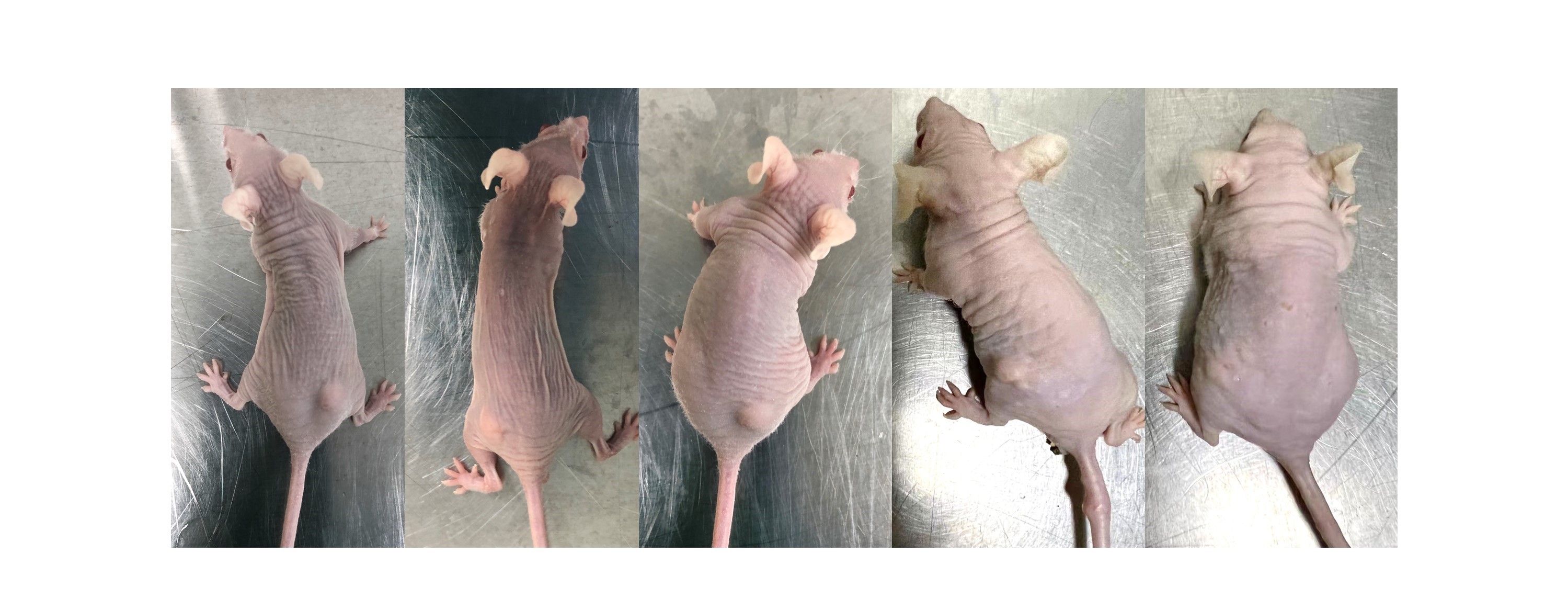

Supplement: Supplementary file 4 — Additional file 4. Figure S3. Representative photos of the animal model. [file 12951_2022_1655_MOESM4_ESM.jpg]
