# Supplementary material for: Exosomal miR-140-5p inhibits osteogenesis by targeting IGF1R and regulating the mTOR pathway in ossification of the posterior longitudinal ligament
Source: J Nanobiotechnology. 2022 Oct 15;20:452. doi: 10.1186/s12951-022-01655-8 (PMC9571456; doi:10.1186/s12951-022-01655-8)
Supplement: Supplementary file 5 — Additional file 5: Table S2. The sequences of qPCR primers. [file 12951_2022_1655_MOESM5_ESM.docx]

**Supplementary Table S2. Characteristics of patients**

| **Group** | **Sex** | **Age** | **Diagnosis** | **Surgical level** |
| --- | --- | --- | --- | --- |
| OPLL 1 | female | 55 | OPLL | C5 |
| OPLL 2 | female | 68 | OPLL | C5 |
| OPLL 3 | male | 60 | OPLL | C3 |
| OPLL 4 | male | 61 | OPLL | C5 |
| OPLL 5 | female | 58 | OPLL | C4 |
| OPLL 6 | female | 61 | OPLL | C4 |
| OPLL 7 | male | 71 | OPLL | C4 |
| OPLL 8 | male | 72 | OPLL | C4 |
| OPLL 9 | female | 62 | OPLL | C3 |
| OPLL 10 | male | 63 | OPLL | C5 |
| OPLL 11 | male | 62 | OPLL | C5 |
| OPLL 12 | female | 70 | OPLL | C5 |
| OPLL 13 | male | 63 | OPLL | C6 |
| OPLL 14 | male | 64 | OPLL | C6 |
| OPLL 15 | male | 56 | OPLL | C4 |
| OPLL 16 | male | 55 | OPLL | C4 |
| OPLL 17 | female | 65 | OPLL | C3-4 |
| OPLL 18 | female | 59 | OPLL | C5-6 |
| OPLL 19 | male | 53 | OPLL | C3 |
| OPLL 20 | male | 54 | OPLL | C4 |
| non-OPLL 1 | female | 48 | cervical spine trauma | C4 |
| non-OPLL 2 | female | 52 | cervical spine trauma | C4 |
| non-OPLL 3 | male | 54 | cervical spine trauma | C4 |
| non-OPLL 4 | female | 57 | cervical spine trauma | C5 |
| non-OPLL 5 | male | 58 | cervical spine trauma | C5 |
| non-OPLL 6 | male | 58 | cervical spine trauma | C5 |
| non-OPLL 7 | male | 62 | cervical spine trauma | C4 |
| non-OPLL 8 | female | 63 | cervical spine trauma | C4 |
| non-OPLL 9 | female | 53 | cervical spine trauma | C3 |
| non-OPLL 10 | female | 54 | cervical spine trauma | C3 |
| non-OPLL 11 | male | 67 | cervical spine trauma | C5 |
| non-OPLL 12 | female | 68 | cervical spine trauma | C6 |
| non-OPLL 13 | male | 52 | cervical spine trauma | C5 |
| non-OPLL 14 | male | 63 | cervical spine trauma | C5 |
| non-OPLL 15 | male | 62 | cervical spine trauma | C4 |
| non-OPLL 16 | female | 63 | cervical spine trauma | C5 |
| non-OPLL 17 | male | 71 | cervical spine trauma | C4 |
| non-OPLL 18 | female | 67 | cervical spine trauma | C4 |
| ***P* value** | **0.745** | **0.307** | **-** | **0.765** |

OPLL: ossification of the posterior longitudinal ligament
